# Examining the role of participant and study partner report in widely-used classification approaches of mild cognitive impairment in demographically-diverse community dwelling individuals: results from the Einstein aging study

**DOI:** 10.3389/fnagi.2023.1221768

**Published:** 2023-11-21

**Authors:** Katherine H. Chang, Cuiling Wang, Caroline O. Nester, Mindy J. Katz, Desiree A. Byrd, Richard B. Lipton, Laura A. Rabin

**Affiliations:** ^1^Department of Psychology, Queens College, City University of New York (CUNY), Queens, NY, United States; ^2^Department of Psychology, The Graduate Center, City University of New York (CUNY), New York, NY, United States; ^3^Department of Epidemiology and Population Health, Albert Einstein College of Medicine, Bronx, NY, United States; ^4^Saul R. Korey Department of Neurology, Albert Einstein College of Medicine, Bronx, NY, United States; ^5^Department of Psychiatry and Behavioral Medicine, Albert Einstein College of Medicine, Bronx, NY, United States; ^6^Department of Psychology, Brooklyn College, City University of New York (CUNY), Brooklyn, NY, United States

**Keywords:** subjective cognitive concerns, mild cognitive impairment, informant report, study partner, aging, longitudinal

## Abstract

**Objective:**

The role of subjective cognitive concerns (SCC) as a diagnostic criterion for MCI remains uncertain and limits the development of a universally (or widely)-accepted MCI definition. The optimal MCI definition should define an at-risk state and accurately predict the development of incident dementia. Questions remain about operationalization of definitions of self- and informant-reported SCCs and their individual and joint associations with incident dementia.

**Methods:**

The present study included Einstein Aging Study participants who were non-Hispanic White or Black, free of dementia at enrollment, had follow-up, and completed neuropsychological tests and self-reported SCC at enrollment to determine MCI status. Informant-reported SCC at baseline were assessed via the CERAD clinical history questionnaire. Self-reported SCC were measured using the CERAD, items from the EAS Health Self-Assessment, and the single memory item from the Geriatric Depression Scale. Cox proportional hazards models examined the association of different operationalizations of SCC with Petersen and Jak/Bondi MCI definitions on the risk of dementia, further controlling for age, sex, education, and race/ethnicity. Time-dependent sensitivity and specificity at specific time points for each definition, and Youden’s index were calculated as an accuracy measure. Cox proportional hazards models were also used to evaluate the associations of combinations of self- and informant-reported SCC with the risk of incident dementia.

**Results:**

91% of the sample endorsed at least one SCC. Youden’s index showed that not including SCC in either Jak/Bondi or Petersen classifications had the best balance between sensitivity and specificity across follow-up. A subset of individuals with informants, on average, had a lower proportion of non-Hispanic Blacks and 94% endorsed at least one self-reported SCC. Both informant-reported and self-reported SCC were significantly associated with incident dementia.

**Conclusion:**

Our findings suggest that the SCC criterion may not improve the predictive validity for dementia when included in widely-employed definitions of MCI. Consistent with some prior research, informant-reported SCC was more related to risk of incident dementia than self-reported SCC. Given that requiring informant report as a diagnostic criterion may unintentionally exclude health disparate groups, additional consideration is needed to determine how best to utilize informant-report in MCI diagnosis.

## 1 Introduction

Mild cognitive impairment (MCI) represents an intermediate stage of cognitive impairment, intended to identify the transitional phase between normal aging and dementia (Petersen et al., 1999; Winblad et al., 2004; Petersen et al., 2014; Scharre, 2019). When the concept of MCI was first introduced, documentation of memory decline was largely drawn from cognitive concerns expressed during the clinical interview, with informant reports of decline and objective neuropsychological data used to corroborate abnormal memory performance (Petersen et al., 1999). Currently, subjective cognitive concern (SCC) is a core criterion for the most widely-used definition of MCI in clinical and research settings (Winblad et al., 2004; Kelley and Petersen, 2007; Albert et al., 2011; Jack et al., 2018; Kasper et al., 2020; Mayo Clinic, 2020; UCSF Weill Institute for Neurosciences, 2022). For other prodromal dementia conditions (e.g., subjective cognitive decline, motoric cognitive risk syndrome), research has examined the assessment of SCC in a more systematic, psychometrically validated manner with some studies using formal SCC screens including cognitive domains beyond memory (i.e., executive functioning, language, attention/concentration, visuospatial navigation, etc.) (Rabin et al., 2020; Diaz-Galvan et al., 2021; Nester et al., 2021; Wasef et al., 2021). However, the measurement of SCC, especially as a criterion for MCI classification, lacks standardization (Stephan et al., 2013; Rabin et al., 2015; Molinuevo et al., 2017).

There are a multitude of studies on mild cognitive impairment (more than 18,000 articles) with highly variable approaches for (1) how to measure SCC (i.e., clinical interview vs. questionnaire; single- vs. multiple-item questionnaire; “homegrown” questionnaires vs. standardized/validated questionnaires; assessing multiple domains vs. only memory; capturing current ability versus change); (2) optimal sources of the SCC report (i.e., self-, informant-, clinician-report); (3) whether corroboration from informant is required; or (4) how to capture and quantify SCC (i.e., any concern, use of a cutoff score or percentage, median or average score, age-appropriate normative scores) (Stephan et al., 2013; Rabin et al., 2015). Further systematic review of how SCC has been measured for the diagnosis of MCI is warranted but beyond the scope of this paper. Supplementary Table 1 provides a sampling to demonstrate the extensive variability in the field but is not intended to be exhaustive. A large proportion of studies list SCC as a criterion for MCI diagnosis, citing Petersen/Winblad’s original definition, but do not provide specific information on how SCC was defined. Some studies do not include SCC as a criterion. Others describe the measure(s) used but do not provide information on the nature of the measurement, source of information, or precise operational definitions of SCC. In studies using SCC questionnaires, items also vary in their referents (relative to previous state personal cognitive status or age-matched mates), time periods (now, over the past year, over the past 5 years), and response option formats (e.g., dichotomous yes/no vs. Likert-like scales for severity, frequency, degree of change).

The original rationale for the inclusion of SCC as a criterion for MCI diagnosis was to identify individuals undergoing cognitive change. Objective cognitive tests measure status, but unless baseline measures are available, they do not capture change. At baseline, cognitive tests may flag individuals with long-standing cognitive difficulties (e.g., learning disabilities, neurodevelopmental disorders, etc.) as impaired (Petersen, 2004). For individuals with above average intellectual abilities, cross-sectional cognitive tests may not capture early stages of cognitive decline if scores fall within normal limits. Despite the compelling rationale, the predictive validity of SCC for incident dementia remains uncertain. For the original Petersen criteria, SCC needed to be self-reported (Petersen et al., 1999), requiring an individual to possess a level of awareness and a willingness to acknowledge memory changes. Though self-reported SCC are associated with objective cognitive performance, they are also associated with depression and anxiety (Buckley et al., 2013; Edmonds et al., 2014; Ryu et al., 2016; Yates et al., 2017; Topiwala et al., 2021; Scholz and Donders, 2022), resulting in overreporting of SCC from cognitively unimpaired individuals (Yates et al., 2017; Edmonds et al., 2018). Many studies show that individuals with MCI demonstrate anosognosia or poor awareness of their own cognitive and functional deficits (Vogel et al., 2004; Edmonds et al., 2014; Fragkiadaki et al., 2016; Gerretsen et al., 2017; Bastin et al., 2021; Ilardi et al., 2021), resulting in underreporting of cognitive changes (Edmonds et al., 2018; Ryu et al., 2019). Further, anosognosia independently predicts conversion from MCI to dementia and is associated with biomarkers, such as reduced brain metabolism (Gerretsen et al., 2017), suggesting that the absence of anosognosia (i.e., insight and SCC) may actually be clinically useful to identify individuals who are unlikely to convert from MCI to dementia (Gerretsen et al., 2017; Bastin et al., 2021). This appears to be contradictory to SCC being a core criterion for defining MCI, and as such, some research efforts support the removal of SCC (Lenehan et al., 2012; Edmonds et al., 2014, 2018; Hackett et al., 2020) on the basis that they do not meaningfully contribute to diagnosis or may even contribute to misdiagnosis of MCI (Vogel et al., 2004; Edmonds et al., 2014; Fragkiadaki et al., 2016; Ilardi et al., 2021). Consistent with this approach and with the various problematic aspects of requiring SCC as a criterion in MCI, Jak, Bondi, and colleagues introduced an approach to MCI classification that uses comprehensive neuropsychological criteria without SCC (Jak et al., 2009; Bondi et al., 2014).

Recent reports suggest that SCC can be assessed by an individual or knowledgeable informant, including clinicians, friends, or family members (Albert et al., 2011; Tangalos and Petersen, 2018). Some research supports that informant-reported SCC or mutual report from both the individual and informant better correlate with an individual’s objective cognitive performance and may better predict progression to dementia than only self-reported SCC (Tierney et al., 1996; Rabin et al., 2012; Gifford et al., 2015; Edmonds et al., 2018; Numbers et al., 2023; Peng et al., 2023). Some investigators address the differences in self- and informant-report with a discrepancy score that may correlate well with objective cognitive impairment (Edmonds et al., 2018) and is a validated approach to measure anosognosia (Starkstein et al., 2006)—which may be clinically useful to improve identification of progression from MCI to dementia. However, informant-reported SCC still has its limitations and can be affected by factors, including type and quality of relationship, frequency of contact, expectations, and affective states of both individual and/or informant (Jessen et al., 2014; Hackett et al., 2020). For example, there may be limited opportunity to notice changes in cognition for older adults who are socially isolated or do not see their families or physicians except for rare occasions.

Whether to include SCC as a criterion for MCI depends critically on its predictive validity for incident dementia. Much of the research on this topic has been carried out in samples of predominantly highly educated, non-Hispanic White older adults, limiting the generalizability of this work to more diverse populations. The current study investigates three operational definitions of self-reported concerns and two definitions of informant-reported concerns about cognition to determine whether their inclusion in two widely-used MCI definitions (i.e., Petersen and Jak/Bondi) add to the predictive validity for incident dementia. Using a more representative cohort of older adults than is typical for aging studies, we additionally investigated discrepancy patterns between self- and informant-reported SCC and their association with incident dementia. Based on prior research (Buckley et al., 2013; Edmonds et al., 2014; Ryu et al., 2016; Yates et al., 2017; Topiwala et al., 2021), we predict that the inclusion of any SCC endorsement (compared to no SCC or a high average cutoff) as a criterion for MCI diagnosis will be the least predictive of incident dementia. Beyond this, there is no prior literature that can specifically guide hypotheses for other operational definitions of SCC as it pertains to the diagnosis of MCI. As such, this work will be exploratory and is an important step forward toward standardizing SCC measurement in MCI.

## 2 Materials and methods

### 2.1 Participants

The EAS is a longitudinal study of community-residing individuals from the Bronx NY, which is a racially and ethnically diverse urban setting (Katz et al., 2012). Details about study recruitment have been described elsewhere (Katz et al., 2012). In brief, participants were systematically recruited using Bronx County Voter Registration lists. Individuals were mailed introductory letters and given a telephone screen to determine study eligibility. Those who met preliminary eligibility criteria were invited for further in-person evaluations. In-person assessments were conducted annually and included comprehensive neurological, medical, psychosocial, and neuropsychological evaluations. All protocols were approved by the Einstein Institutional Review Board (IRB) and written informed consent was obtained at the initial clinic visit. Inclusion criteria were age 70 and above, resident of Bronx, NY, noninstitutionalized, and English speaking. Exclusion criteria at baseline included severe audiovisual, physical impairments, or active psychiatric symptomatology, which may interfere with the ability to complete assessments (Katz et al., 2012). Participants eligible for the present study were enrolled between October 1993 and June 2016, had at least one annual follow-up, and were free of dementia at baseline and first follow-up visit.

### 2.2 Measures

#### 2.2.1 Neuropsychological assessment

Participants completed standardized neuropsychological testing at baseline and all annual follow-up visits (Katz et al., 2012). Five cognitive domains were used for MCI diagnosis: memory, attention, executive functioning, language, and visuospatial functioning, with two tests included in each domain. The memory domain included the Free and Cued Selective Reminding Test (FCSRT) (Buschke, 1984) and the Wechsler Memory Scale-Revised Logical Memory I subtest (WMS-R-LMI) (Wechsler, 1987). Attention/processing speed was measured using the Digit Span subtest of the Wechsler Adult Intelligence Scale-III (Wechsler, 1997) and the Trail Making Test, part A (Reitan, 1958). Executive functioning tests included Trail Making Test, part B (Reitan, 1958) and the Letter Fluency “FAS” task (Spreen and Strauss, 2006). Language was measured with the Category Fluency task (animals, vegetables, fruits) (Rosen, 1980) and the Boston Naming Test (Kaplan et al., 1983). Visuospatial functioning tests included the Block Design and Digit Symbol subtest from the WAIS-III (Wechsler, 1997).

Normative data were calculated using local norms derived by cognitively unimpaired individuals in the sample. Participants were classified as CU if they had: (1) no significant SCC, measured by no endorsement of any item on three SCC questionnaires (detailed below); (2) unimpaired self-reported ADL as measured by the IADL Lawton Brody Scale (Lawton and Brody, 1969); and (3) cognitive functioning within normal limits as defined by having two out of three of the following at baseline: (i) a global score of 0 (“normal cognition”) on the CDR^®^ Dementia Staging Instrument (CDR^®^, Morris, 1997), (ii) a score of 3 or lower on the Blessed Information–Memory–Concentration Test (BIMC; Blessed et al., 1968), or (iii) a score of 5 or greater on the Memory Impairment Screen (MIS; Buschke et al., 1999).

#### 2.2.2 Subjective cognition assessment

##### 2.2.2.1 Self

Twenty-two items derived from three questionnaires were used to assess self-perceived cognitive functioning: 17 items from the CERAD clinical history questionnaire (Morris et al., 1989), a yes/no/do not know rating scale of current functioning in several cognitive domains; four items from the EAS Health Self-Assessment (HSA; Derby et al., 2013) that inquired about current memory problems and changes in memory compared to 1 to 10 years prior to the baseline assessment (ordinal data 3 to 4 response options); and the dichotomous memory item from the short form of the Geriatric Depression Scale (GDS): “Do you feel you have more problems with your memory than most?” (Sheikh and Yesavage, 1986).

##### 2.2.2.2 Informant

Informant perceptions of participants’ cognition were collected from 17 corresponding items from the CERAD (informant form), a yes/no/do not know rating scale of participants’ current cognitive functioning.

### 2.3 MCI classification and operationalization of SCC

MCI classifications were made using two widely-used MCI criteria: (1) updated Petersen criteria (Winblad et al., 2004; Artero et al., 2006) requiring (a) objective memory impairment (>1.5 SD below the age-, sex-, education-, and race/ethnicity-adjusted mean); (b) subjective memory impairment operationalized as any SCC indicated by self- or informant-report (measures discussed below); (c) absence of functional decline as measured by the IADL Lawton Brody scale (Lawton and Brody, 1969); and (d) no diagnosis of dementia and (2) Jak/Bondi comprehensive neuropsychological criteria (Bondi et al., 2014) requiring (a) one low score (>1 SD below the age-, sex-, education-, and race/ethnicity-adjusted mean) on both measures within at least one cognitive domain; or (b) at least one low score (>1 SD below the age-, sex-, education-, and race/ethnicity-adjusted mean) across at least two cognitive domains.

Table 1 depicts the operational definitions of SCC that were included as a criterion in MCI classifications. The definitions using self-reported SCC were as follows: (1) no inclusion of SCC; (2) any concern (at least one SCC question endorsed); and (3) a high average cutoff (Q3) of concern. Two definitions of informant-reported SCC were as follows: (1) any informant-reported concern (at least one item on the informant CERAD questionnaire was endorsed) and (2) a high average cutoff (Q3) on the informant CERAD. Of note, because Petersen MCI classification already contains a criterion for SCC, this criterion was removed for the “no inclusion of SCC” condition.

**Table 1:** Operational definitions of self-reported SCC.

Jak/Bondi	
with no SCC	SCC criterion not included, cognitive tests only
+ any self-reported SCC	At least one SCC question endorsed*
+ high avg. self-reported SCC + any informant-reported SCC + high avg. informant-reported SCC	High average cutoff (Q3) of all SCC questionnaires At least one SCC question endorsed on the CERAD High average cutoff (Q3) on the CERAD
Petersen	
with no SCC	SCC criterion removed, cognitive tests only
+ any self-reported SCC	At least one SCC question endorsed*
+ high average self-reported SCC + any informant-reported SCC + high avg. informant-reported SCC	High average cutoff of all SCC questionnaires At least one SCC question endorsed on the CERAD High average cutoff (Q3) on the CERAD

* Based on all self-reported SCC questionnaires including CERAD, HSA, and the memory item on the GDS.

An additional analysis was conducted with individuals with informants to examine the discrepancy between self-reported and informant-reported SCC using the CERAD questionnaire. Using low levels (i.e., below the cut-off of Q3) of self- or informant-reported SCC as reference, the following combinations were used to reflect patterns of discrepancy: (1) high levels of both self- and informant-reported SCC; (2) high levels of informant-reported SCC and low self-reported SCC; and (3) high levels of self-reported SCC and low informant-reported SCC (Table 2).

**Table 2:** Combinations of self- and informant-report on the CERAD questionnaire.

		Informant	
		Low level of SCC report	High level of SCC report
Self	Low level of SCC report	Neither informant nor participant endorse a high amount of SCC	Informant reports a high amount of SCC but participant does not
	High level of SCC report	Participant reports a high amount of SCC but their informant does not	Both informant and participant endorse a high amount of SCC

#### 2.3.1 Dementia

Incident dementia diagnosis at follow-up was the main outcome. Broadly consistent with DSM-IV for major neurocognitive disorder (American Psychiatric Association, 1994), participants were classified as having incident dementia if all of the following criteria are met: (1) there was substantial cognitive impairment on objective measures—that is, scores at least 1.5 standard deviations below the age-adjusted mean; (2) the participant or study informant reported changes in cognitive function; (3) there was functional decline determined at a case conference based on information from self or informant report, impairment scores on the IADL Lawton Brody Scale (Lawton and Brody, 1969), and clinical evaluation; and (4) cognitive impairment was not better explained by the effects of a substance or medication.

### 2.4 Demographic and clinical characteristics

Demographic information from the EAS included self-reported race/ethnicity as defined by the U.S. Census Bureau in 1994 (categorized to: non-Hispanic White, non-Hispanic Black), number of years of education, sex, and age. Subclinical symptoms of depression were assessed using the GDS short form, excluding the single memory item (Sheikh and Yesavage, 1986).

### 2.5 Data analysis

The time to event was defined as the time between the baseline clinic visit and the date of dementia diagnosis or the final follow-up visit when the participants were known without dementia. To evaluate the associations of five operationalizations of SCC into the MCI definitions at baseline with the risk of incident dementia, Cox proportional hazards models were applied. Because the cumulative dementia disease status is time-dependent and time to dementia can be censored, time-dependent receiver operating characteristic (ROC) based on the Cox model was used to evaluate the discriminative ability of each MCI for dementia incidence within a given time period. Specifically, the time-dependent sensitivity and specificity for cumulative disease incidence within a time interval t were defined as


$$Se\left(t\right)=P\left(X=1|T\leq t\right)=\frac{\left(1-S\left(t|X=1\right)\right)P\left(X=1\right)}{1-S\left(t\right)}$$

$$Sp\left(t\right)=P\left(X=0|T>t\right)=\frac{S\left(t|X=0\right)P\left(X=0\right)}{S\left(t\right)}$$

Where X is MCI status at baseline, T is time to dementia, S(t|X=x), x =0,1, is the conditional survival probability given *X*, S(t)=S(t|X=1)P(X=1)+S(t|X=0)P(X=0) is the overall survival probability. They were estimated using estimates of the survival probabilities from the Cox model and estimate of the prevalence of MCI. Youden’s index, Se(t)+Sp(t)−1, was then obtained as a compromised accuracy measure. Results at 2-, 3-, 5-, and 7-years follow-up, along with the numbers of participants at risk at the time, were reported.

Additional models controlling for covariates including age, sex, education, race/ethnicity, and depressive symptoms at baseline were also applied. Cox proportional hazards models were also used to evaluate the associations of combinations of self- and informant-reported SCC with the risk of incident dementia. All statistical analyses were performed using SAS 9.4 (SAS Institute Inc., Cary, N.C.).

## 3 Results

### 3.1 Overview

At baseline (*N* = 1,097), participants’ age ranged from 70 to 100 (mean = 78.6 ± 5.4) years, the sample was 62.4% female and educational achievement averaged 13.7 ± 3.5 years, with 45.1% obtaining 12 years or fewer years of education. Notably, nearly one fifth of participants (18.9%) did not complete high school, highlighting the educational diversity within the sample. Most participants identified as White (70.6%), though Black participants were well-represented (29.4%). During up to 19.6 years of follow-up (mean 4.5 years, median 3.3 years), 124 individuals developed incident dementia. As shown in Table 3, those who developed dementia during follow-up were significantly older and had fewer years of education at baseline. The groups did not differ in number of depressive symptoms, sex, race/ethnicity, or follow-up time.

**Table 3:** Baseline descriptive characteristics of the sample by dementia status.

	All^^^	No dementia during follow-up	Incident dementia	*p*
Sample Size: #, %	1,097	973, 88.7%	124, 11.3%	
Age at baseline: *M (SD)*	78.6 (5.4)	78.2 (5.3)	81.0 (5.2)	<0.0001
Sex: #, % Female	684, 62.4%	604, 62.1%	80, 64.5%	0.597
Education: *M (SD)*	13.7 (3.5)	13.8 (3.5)	13.1 (3.6)	0.046
*Race/Ethnicity:*				
#, % non-Hispanic White	775, 70.6%	692, 71.1%	83, 66.9%	0.335
#, % non-Hispanic Black	322, 29.4%	281, 28.9%	41, 33.1%	
GDS score^+^: *M (SD)*	2.2 (2.3)	2.2 (2.3)	2.5 (2.4)	0.089
Any self-reported SCC: %	91.3%	91.4%	91.1%	0.929
*MCI status: #, %*				
Jak/Bondi with no SCC	422, 38.5%	334, 34.3%	88, 71.0%	<0.0001
Jak/Bondi + any self-reported SCC	375, 34.2%	296, 30.4%	79, 63.7%	<0.0001
Jak/Bondi + high avg. self-reported SCC	162, 14.8%	114, 11.7%	48, 38.7%	<0.0001
Petersen with no SCC	555, 50.6%	452, 46.5%	103, 83.1%	<0.0001
Petersen + any self-reported SCC	494, 45.0%	402, 41.3%	92, 74.2%	<0.0001
Petersen + high avg. self-reported SCC	209, 19.1%	154, 15.8%	55, 44.4%	<0.0001
Follow-up time, years: Median (IQR)	3.2 (2.0–6.2)	3.3 (2.0–6.3)	3.1 (1.7–6.1)	0.456

^ Participants free of prevalent dementia at baseline who had at least one follow-up evaluation and completed neuropsychological tests and self-reported SCC at enrollment.

+ excluding the memory item from the short form of the GDS.

### 3.2 Relationship of self- and informant-reported SCC and risk of incident dementia

Table 4 shows the baseline descriptive characteristics for individuals with informants versus no informants. Individuals with informants, on average, had a lower proportion of non-Hispanic Blacks (25.6% versus 35.4%, *p* = 0.001) and a higher proportion endorsed at least one self-reported SCC (93.9% versus 87.2%, *p* < 0.001). Among those with informants, high average cutoff for informant-reported SCC (HR = 2.52, *p* = 0.003) was slightly more strongly related to incident dementia than self-reported SCC using the same questionnaire (HR = 2.03, *p* = 0.005), but both were significantly associated, controlling for baseline age, sex, education, and race/ethnicity (Table 5).

**Table 4:** Baseline descriptive characteristics of the sample by informant status.

	No informant	Yes informant	*p*
Sample Size: #, %	421, 38.4%	676, 61.6%	
Age at baseline: *M (SD)*	78.6 (5.3)	78.5 (5.4)	0.620
Sex: #, % Female	270, 64.1%	414, 61.2%	0.337
Education: *M (SD)*	13.6 (3.5)	14.0 (3.5)	0.140
*Race/Ethnicity:*			
#, % non-Hispanic White	272, 64.6%	503, 74.4%	0.001
#, % non-Hispanic Black	149, 35.4%	173, 25.6%	
GDS score^+^: *M (SD)*	2.4 (2.6)	2.1 (2.1)	0.421
Any self-reported SCC: %	87.2%	93.9%	0.0001
*MCI status: #, %*			
Jak/Bondi with no SCC	193, 45.8%	229, 33.9%	<0.0001
Jak/Bondi + any self-reported SCC	159, 37.8%	216, 32.0%	0.048
Jak/Bondi + high avg. self-reported SCC	61, 14.5%	101, 14.9%	0.8376
Petersen with no SCC	249, 59.1%	306, 45.3%	<0.0001
Petersen + any self-reported SCC	218, 51.8%	276, 40.8%	0.0004
Petersen + high avg. self-reported SCC	75, 17.8%	134, 19.8%	0.410

+ excluding the memory item from the short form of the GDS.

**Table 5:** Estimates of associations of self-reported SCC and informant-reported SCC on incident dementia, adjusting for age, education, sex, and race/ethnicity.

	Hazard Ratio	95% CI	p*
Age	1.13	(1.08, 1.18)	<0.0001
Sex (Male reference)	1.20	(0.72, 2.00)	0.488
Education, years	0.95	(0.89, 1.03)	0.196
Race/Ethnicity (NH-Black vs. White)	0.95	(0.53, 1.69)	0.850
High level of self-reported SCC	2.03	(1.23, 3.33)	0.005
High level of informant-reported SCC	2.52	(1.53, 4.15)	0.0003

Table 6 shows the association of combined categories of having high levels of self- and informant-report SCC (using the same questionnaire) with risk of dementia. Using low self- and informant-reported SCC as the reference, the risk of dementia was higher among those with high informant-reported SCC and high self-reported SCC (HR = 5.14, *p* < 0.0001) and those with high informant-reported SCC but low self-reported SCC (HR = 2.32, *p* = 0.017). The risk of dementia was also higher, though not significant, in those with high self-reported SCC but low informant-reported SCC (HR = 1.85, *p* = 0.086).

**Table 6:** Estimates of combinations of self-reported SCC and informant-reported SCC on incident dementia, adjusting for age, education, sex, and race/ethnicity.

	Hazard Ratio	95% CI	p*
Age	1.13	(1.08, 1.18)	<0.0001
Sex (Female vs. male)	1.18	(0.71, 1.98)	0.513
Education, years	0.95	(0.89, 1.02)	0.185
Race/Ethnicity (NH- Black vs. White)	0.95	(0.53, 1.70)	0.858
High informant- and high self-reported SCC	5.14	(2.78, 9.51)	<0.0001
High informant- and low self-reported SCC	2.32	(1.16, 4.63)	0.017
Low informant- but high self-reported SCC	1.85	(0.92, 3.75)	0.086

Reference group: Low levels of self- and informant-reported SCC.

### 3.3 Time dependent ROC of MCI defined with and without SCC ascertained from the participant

Notably, 91% of the entire sample endorsed at least one self-reported SCC. Comparisons of time-dependent ROC results for various operational definitions of self-reported SCC (not including SCC, any SCC, and high average, Q3 cutoff) as a criterion in both Jak/Bondi and Petersen MCI classifications, at 2-, 3-, 5-, and 7-years of follow-up and numbers of participants at risk at the time are shown in Tables 7, 8, respectively. Youden’s index, which is determined by the sum of sensitivity and specificity, was highest in all cases for definitions that did not include SCC for both the Jak/Bondi and the Petersen classifications after 2-, 3-, 5-, and 7-years of follow-up. Though inclusion of SCC improved specificity, those gains were more than offset by loss of sensitivity.

**Table 7:** Sensitivity, specificity, and Youden’s index for various operationalization of self-reported SCC in Jak/Bondi MCI at baseline for dementia after 2 to 7 years of follow-up.

Follow-up year (# of participants)	SCC definition	Sensitivity	Specificity	Youden’s index
2 (800)	Jak/Bondi with no SCC	0.767	0.631	0.397
	Jak/Bondi + any self-reported SCC	0.703	0.673	0.376
	Jak/Bondi + high avg. self-reported SCC	0.471	0.865	0.336
3 (615)	Jak/Bondi with no SCC	0.762	0.644	0.406
	Jak/Bondi + any self-reported SCC	0.697	0.685	0.383
	Jak/Bondi + high avg. self-reported SCC	0.459	0.876	0.335
5 (381)	Jak/Bondi with no SCC	0.753	0.665	0.419
	Jak/Bondi + any self-reported SCC	0.688	0.705	0.393
	Jak/Bondi + high avg. self-reported SCC	0.441	0.892	0.333
7 (233)	Jak/Bondi with no SCC	0.729	0.691	0.420
	Jak/Bondi + any self-reported SCC	0.395	0.949	0.344
	Jak/Bondi + high avg. self-reported SCC	0.743	0.690	0.433

**Table 8:** Sensitivity, specificity, and Youden’s index for various operationalization of self-reported SCC in Petersen MCI at baseline for dementia after 2 to 7 years of follow-up.

Follow-up year (# of participants)	SCC definition	Sensitivity	Specificity	Youden’s index
2 (800)	Petersen with no SCC	0.861	0.508	0.369
	Petersen + any self-reported SCC	0.787	0.563	0.351
	Petersen + high avg. self-reported SCC	0.491	0.821	0.312
3 (615)	Petersen with no SCC	0.858	0.521	0.379
	Petersen + any self-reported SCC	0.783	0.575	0.359
	Petersen + high avg. self-reported SCC	0.482	0.831	0.314
5 (381)	Petersen with no SCC	0.853	0.541	0.394
	Petersen + any self-reported SCC	0.777	0.594	0.371
	Petersen + high avg. self-reported SCC	0.469	0.846	0.316
7 (233)	Petersen with no SCC	0.848	0.564	0.411
	Petersen + any self-reported SCC	0.770	0.615	0.385
	Petersen + high avg. self-reported SCC	0.456	0.861	0.317

### 3.4 Time dependent ROC of MCI definition with and without SCC ascertained from the participant or informant

In the subset of the sample with informants available, 94% endorsed at least one self-reported SCC. Comparisons of time-dependent ROC results for various operational definitions of self-reported SCC (not including SCC, any SCC, and high average cutoff) and informant-reported SCC (any SCC and high-average cutoff) as a criterion in both Jak/Bondi and Petersen MCI classifications are shown in Tables 9, 10, respectively. Youden’s index was highest for definitions that included any self-reported SCC for both the Jak/Bondi and the Petersen classifications after 2-, 3-, 5-, and 7-years of follow-up, followed very closely by definitions that did not include any SCC. Notably, the difference between Youden’s indices for these two definitions was less than 0.008 or less, suggesting minimal benefit of including SCC compared to no inclusion. Though inclusion of informant-reported SCC improved specificity, the gains were again more than offset by the drastically decreased sensitivity.

**Table 9:** Sensitivity, specificity, and Youden’s index for various operationalization of self- and informant-reported SCC in Jak/Bondi MCI at baseline for dementia after 2 to 7 years of follow-up.

Follow-up year (# of participants)	SCC definition	Sensitivity	Specificity	Youden’s index
2 (501)	Jak/Bondi with no SCC	0.746	0.675	0.421
	Jak/Bondi + any self-reported SCC	0.734	0.694	0.429
	Jak/Bondi + high avg. self-reported SCC	0.514	0.863	0.377
	Jak/Bondi + any informant-reported SCC	0.528	0.822	0.350
	Jak/Bondi + high avg. informant-reported SCC	0.497	0.902	0.399
3 (382)	Jak/Bondi with no SCC	0.740	0.688	0.428
	Jak/Bondi + any self-reported SCC	0.728	0.708	0.436
	Jak/Bondi + high avg. self-reported SCC	0.502	0.874	0.376
	Jak/Bondi + any informant-reported SCC	0.519	0.832	0.352
	Jak/Bondi + high avg. informant-reported SCC	0.480	0.914	0.394
5 (244)	Jak/Bondi with no SCC	0.731	0.708	0.440
	Jak/Bondi + any self-reported SCC	0.718	0.728	0.447
	Jak/Bondi + high avg. self-reported SCC	0.483	0.891	0.374
	Jak/Bondi + any informant-reported SCC	0.505	0.848	0.353
	Jak/Bondi + high avg. informant-reported SCC	0.452	0.931	0.383
7 (145)	Jak/Bondi with no SCC	0.720	0.732	0.453
	Jak/Bondi + any self-reported SCC	0.706	0.753	0.459
	Jak/Bondi + high avg. self-reported SCC	0.462	0.908	0.370
	Jak/Bondi + any informant-reported SCC	0.488	0.866	0.355
	Jak/Bondi + high avg. informant-reported SCC	0.421	0.948	0.369

**Table 10:** Sensitivity, specificity, and Youden’s index for various operationalization of self-and informant-reported SCC in Petersen MCI at baseline for dementia after 2 to 7 years of follow-up.

Follow-up year (# of participants)	SCC definition	Sensitivity	Specificity	Youden’s index
2 (501)	Petersen with no SCC	0.829	0.560	0.389
	Petersen + any self-reported SCC	0.793	0.605	0.398
	Petersen + high avg. self-reported SCC	0.524	0.813	0.337
	Petersen + any informant-reported SCC	0.588	0.751	0.339
	Petersen + high avg. informant-reported SCC	0.505	0.870	0.375
3 (382)	Petersen with no SCC	0.825	0.572	0.397
	Petersen + any self-reported SCC	0.789	0.617	0.406
	Petersen + high avg. self-reported SCC	0.516	0.823	0.339
	Petersen + any informant-reported SCC	0.582	0.761	0.342
	Petersen + high avg. informant-reported SCC	0.492	0.881	0.374
5 (244)	Petersen with no SCC	0.820	0.590	0.410
	Petersen + any self-reported SCC	0.783	0.636	0.418
	Petersen + high avg. self-reported SCC	0.504	0.837	0.341
	Petersen + any informant-reported SCC	0.572	0.776	0.348
	Petersen + high avg. informant-reported SCC	0.473	0.897	0.371
7 (145)	Petersen with no SCC	0.813	0.613	0.426
	Petersen + any self-reported SCC	0.775	0.658	0.433
	Petersen + high avg. self-reported SCC	0.490	0.853	0.343
	Petersen + any informant-reported SCC	0.560	0.794	0.353
	Petersen + high avg. informant-reported SCC	0.452	0.914	0.366

## 4 Discussion

Whether or not to include SCC as a criterion in the classification of MCI is an uncertainty in the field—but one with important implications. A research-driven approach to the inclusion, exclusion, and/or specific operationalization would be ideal to address this question; however, relatively little attention has been given to standardizing and optimizing the SCC criterion. Moreover, research examining MCI criteria in diverse and/or health disparate populations is limited, which not only restricts the generalizability of the construct, but also upholds health inequities in aging populations (Turner et al., 2022). The present study investigated three operational definitions of self-reported SCC and two operational definitions of informant-reported SCC, within the classification of MCI, in a demographically diverse cohort of older adults. Overall, results indicated that not including self-reported SCC in either Jak/Bondi or Petersen MCI classifications provided the best balance between sensitivity and specificity for incident dementia, across 2-, 3-, 5-, and 7-years of follow-up. In a subset of the sample that had informants available, not including self-reported SCC was comparable to definitions that included any self-reported SCC.

Results are consistent with a growing literature that has questioned the utility of SCC as a criterion in MCI classification, primarily on the basis that SCC may contribute to the misdiagnosis of MCI (Mitchell, 2008; Lenehan et al., 2012; Edmonds et al., 2014; Yates et al., 2017; Edmonds et al., 2018). Self-perceived SCC are present across the lifespan and typically increase with age, with approximately 11% of adults aged 45–64 years endorsing SCC and growing to 50–80% of those over the age of 70 (Mitchell, 2008; Taylor et al., 2018; CDC, 2019; Jessen et al., 2020). Extensive variability in methods of SCC assessment for aging populations (see Stephan et al., 2013; Rabin et al., 2015; Molinuevo et al., 2017) likely contributes to the difference in the prevalence of SCC between studies and highlights the need for further research toward standardizing SCC measurement. In addition to inconsistency in operationalization, there can be challenges in disentangling the numerous demographic, psychosocial, and health related factors that complicate the relationship between SCC, objective cognition, and neurodegeneration. Emerging research shows inconsistent associations between SCC and objective memory in racial/ethnic minoritized individuals (Sims et al., 2011; Jackson et al., 2017; Hughes et al., 2020; Rodriguez et al., 2021; Chapman et al., 2023; Katz et al., 2023). SCC reporting may differ (1) among racial/ethnic groups; (2) among those with different levels of formal educational attainment; or (3) due to sociocultural factors that impact the perception subjective experience, and meaning of cognitive changes (Sayegh and Knight, 2013; CDC, 2019; Hughes et al., 2020; Dilworth-Anderson and Gibson, 2022; Katz et al., 2023). Overall, additional research investigating how cultural factors such as level of education, language status, or cultural norms and values impact an individual’s understanding of their own cognitive status and how they endorse cognitive concerns. These cultural differences provide an added layer of complexity to measuring SCC in diverse individuals and using it as a reliable criterion for MCI.

Further contributing to the instability of SCC as a diagnostic criterion, cognitive concerns may evolve as a result of biopsychosocial variables, such as mood, somatic conditions, chronic pain, medication use, sleep disorders, psychosocial stress, personality factors, and/or normal aging (Bassett and Folstein, 1993; Comijs et al., 2002; Mitchell, 2008; Elfgren et al., 2010; Yates et al., 2017; Jessen et al., 2020; Jenkins et al., 2021; Scholz and Donders, 2022) and resolution of these factors may impact the course of SCC over time (Glodzik-Sobanska et al., 2007; Vestberg et al., 2010). On the other hand, subsets of older adults who endorse persistent SCC but do not show cognitive impairment on sensitive neuropsychological measures (i.e., do not meet criteria for MCI) are more likely to demonstrate objective cognitive decline in the future and are at higher risk for ultimately developing dementia (Treves et al., 2005; Jessen et al., 2014; Luck et al., 2015; Molinuevo et al., 2017; Rabin et al., 2017). It is notable, however, that many individuals who meet criteria for subjective cognitive decline (SCD), do not ultimately progress to dementia (Jessen et al., 2020) and the association of SCD with incident MCI is also not clearly understood (Warren et al., 2022). In the clinical context, a careful evaluation of SCC is essential to identify and treat any plausibly modifiable contributors and to identify older adults in need of additional work-up. Thus, as a clinical tool, SCC may be valuable during preclinical phases (i.e., when an individual’s insight remains intact and prior to objective cognitive impairment) or when the clinical question is to rule out dementia (i.e., when increased specificity is needed). However, in research settings or when individuals progress to more overt cognitive impairment, the complex, multifactorial contributors to SCC may cloud the predictive validity for dementia when SCC is included in MCI definitions.

Although the association between SCC and dementia risk is complex, our results showed both self- and informant-reported SCC was related to risk of dementia. Additionally, the risk of dementia was significantly higher among those with high levels of informant-reported SCC, regardless of the level of self-reported SCC, but the risk of dementia was not as high in those with high levels of self-reported SCC but low informant-reported SCC. This corroborates previous research showing that informant-reported SCC or mutual report better correlate with an individual’s objective cognitive performance and better predicts progression to dementia than only self-reported SCC (Tierney et al., 1996; Rabin et al., 2012; Gifford et al., 2015; Edmonds et al., 2018; Peng et al., 2023). Self-awareness of cognitive deficits and the discrepancy between self- and informant-report have a U-shaped distributions. Self-reported cognitive concerns tend to be elevated relative to informant-reported concerns in SCD (i.e., high discrepancy, Rami et al., 2014; Ryu et al., 2019), become more aligned in early MCI (i.e., low discrepancy, Rabin et al., 2017; Edmonds et al., 2018; Ryu et al., 2019), and become more discrepant as anosognosia develops in late MCI and dementia (Edmonds et al., 2014; Rueda et al., 2014; Rabin et al., 2017). Given the possible fluctuation in self-reported SCC and self-awareness of cognitive and adaptive functioning, this may explain why utilizing SCC was not additive to predicting incident dementia in our sample.

Importantly, although collateral information about an older adult’s daily functioning can be of great benefit in the clinical setting, requiring the presence of an informant may, in fact, hamper ADRD research efforts. For example, excluding older adults without informants effectively translates to excluding socially isolated older adults, who may be at greater risk for dementia (Huang et al., 2023). In addition to having more social support, older adults who have available informants may be demographically different in other ways (e.g., fewer medical comorbidities, better quality of life, etc., Cacioppo and Cacioppo, 2013; Portacolone et al., 2018). Of note, the EAS recruits from the Bronx, NY, a county with one of the most diverse U.S.-born older adult populations in New York and some of the poorest health metrics. Only 62% of our sample had informants available, and this subset had a higher proportion of non-Hispanic Whites, suggesting an unintended selection bias for studies that exclude those without study partners. To promote health equality, it is necessary to examine diagnostic criteria and direct research efforts to capture the intended at-risk populations.

Several study limitations warrant mention. Only a small subset of participants was joined by their informants, and there was a limited number of incident dementia cases. Additional studies in larger samples are needed. Although non-Hispanic Blacks were well represented, the population of Hispanic older adults or those of other racial/ethnic groups were insufficient to include in the analyses. Additionally, the self- and informant-report SCC questionnaires were limited in their coverage of cognitive domains and not all measures were available for both participants and their informants. As EAS has evolved, we have given much more consideration to the measurement of SCC and have newly implemented an expanded version of the widely-used Cognitive Change Index (CCI-40, Nester et al., 2022). Future research could expand on this current study utilizing standardized measures of SCC and exploring their utility in other health disparate groups.

To our knowledge, this was the first longitudinal study directly comparing five specific operational definitions of self- or informant-reported SCC in a diverse community-based sample. Our findings suggest that not including SCC, across different MCI definitions, provides the best balance of sensitivity and specificity in terms of predicting incident dementia in our sample of diverse individuals. Although the original rationale was to avoid prescribing a diagnosis of MCI to individuals with long-standing cognitive difficulties (e.g., learning disabilities) (Petersen, 2004), the extremely high prevalence of self-reported SCC in our sample warrants consideration as to whether this criterion is diagnostically additive and/or should be removed from MCI classifications. Consistent with prior research, informant-reported SCC was more strongly related to risk of incident dementia than self-perceived cognitive concerns. However, requiring informant reports in diagnostic criteria for MCI or as inclusion criteria in research settings may unintentionally exclude health disparate groups and/or those at elevated risk for dementia. Additional consideration is needed to determine how best to utilize informant-report in MCI screening and diagnosis. Taken together, these results provide valuable information for researchers and clinicians in their understanding of self- and informant-reported SCC and their relation to MCI classifications and incident dementia in diverse community-based populations. Importantly, this research can serve as a springboard for future efforts (e.g., pooling data) and to stimulate increased discussion among experts to arrive at an optimal MCI definition that promotes diagnostic accuracy while also being mindful of factors that may uphold health inequalities in aging populations.

## Data availability statement

The terms of consent for research participants stipulate that an individual’s data can only be shared outside of the EAS investigators group if the group has reviewed and approved the proposed secondary use of the data. This consent applies regardless of whether data has been de-identified. Access is mediated via a standardized request process via Mindy Katz, mindy.katz@einsteinmed.edu.

## Ethics statement

All protocols were approved by the Einstein Institutional Review Board (IRB). The studies were conducted in accordance with the local legislation and institutional requirements. Written informed consent from the participants or the participants’ legal guardians/kin was obtained at the initial clinic visit.

## Author contributions

KC and LR are the corresponding authors and guarantors of this manuscript and contributed to the conception and the design of the study. MK and CW organized the database. CW performed the statistical analysis and assisted with interpretation of results. KC, in collaboration with the co-authors, wrote the first draft of the manuscript, incorporated all levels of feedback from co-authors, and prepared the manuscript for submission. RL, DB, and CN provided expert guidance and reviewed this manuscript and revisions. The project was funded by successful project grants secured by MK and RL. All authors contributed to manuscript revision, read, and approved the submitted version.
