# Supplementary material for: Examining the role of participant and study partner report in widely-used classification approaches of mild cognitive impairment in demographically-diverse community dwelling individuals: results from the Einstein aging study
Source: Front Aging Neurosci. 2023 Nov 21;15:1221768. doi: 10.3389/fnagi.2023.1221768 (PMC10702963; doi:10.3389/fnagi.2023.1221768)
Supplement: Supplementary file 1 [file Image_1.PDF]

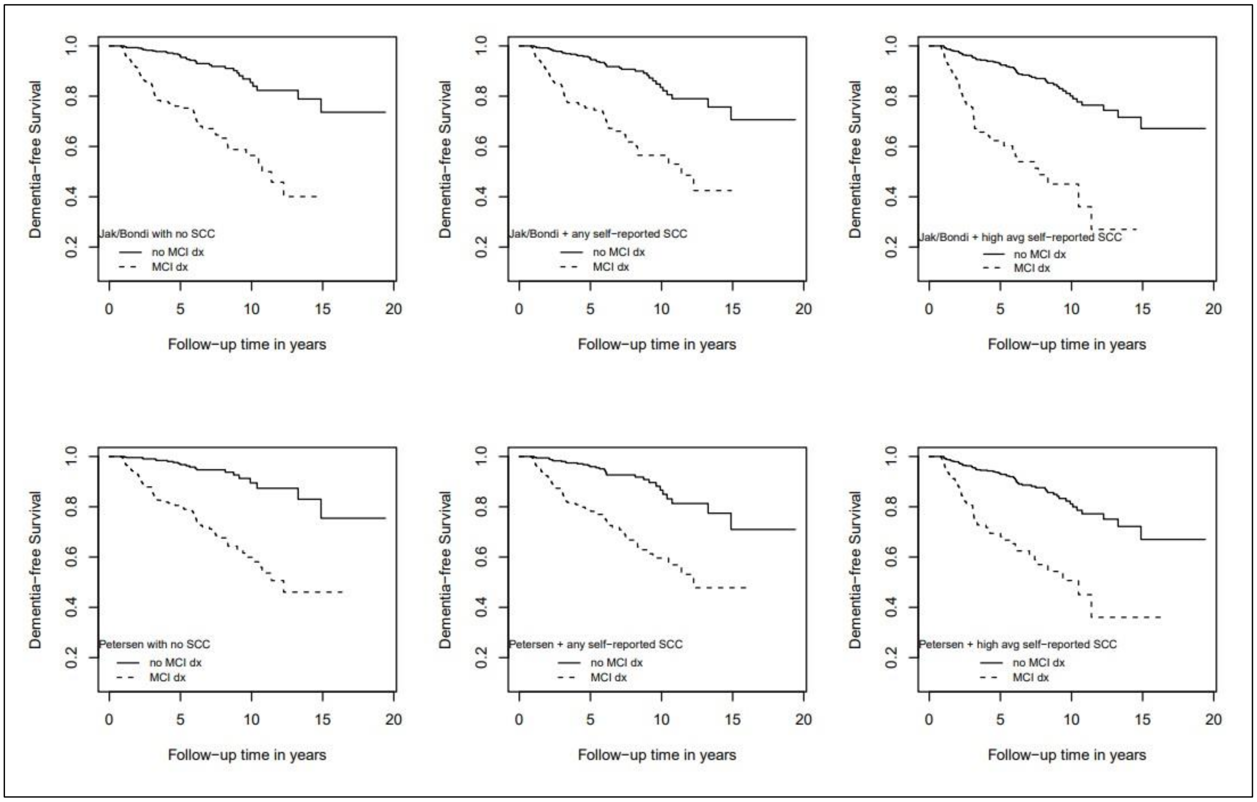

Supplementary Figure 1. Kaplan-Meier survival curves for incident dementia by baseline MCI status using each operational definition of SCC.

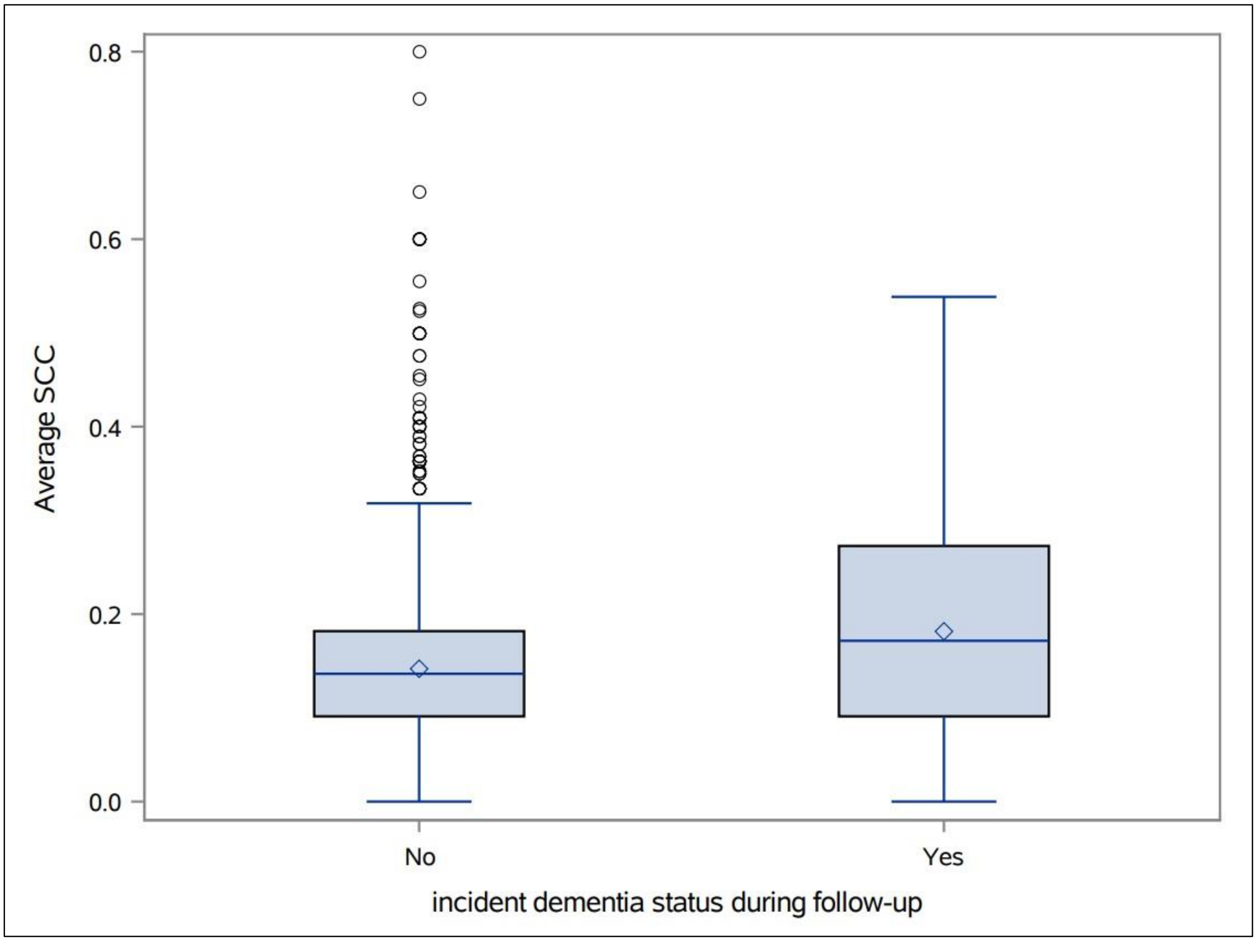

Supplementary Figure 2. Box plot of average self-reported SCC by incident dementia status at follow-up in the entire study sample.

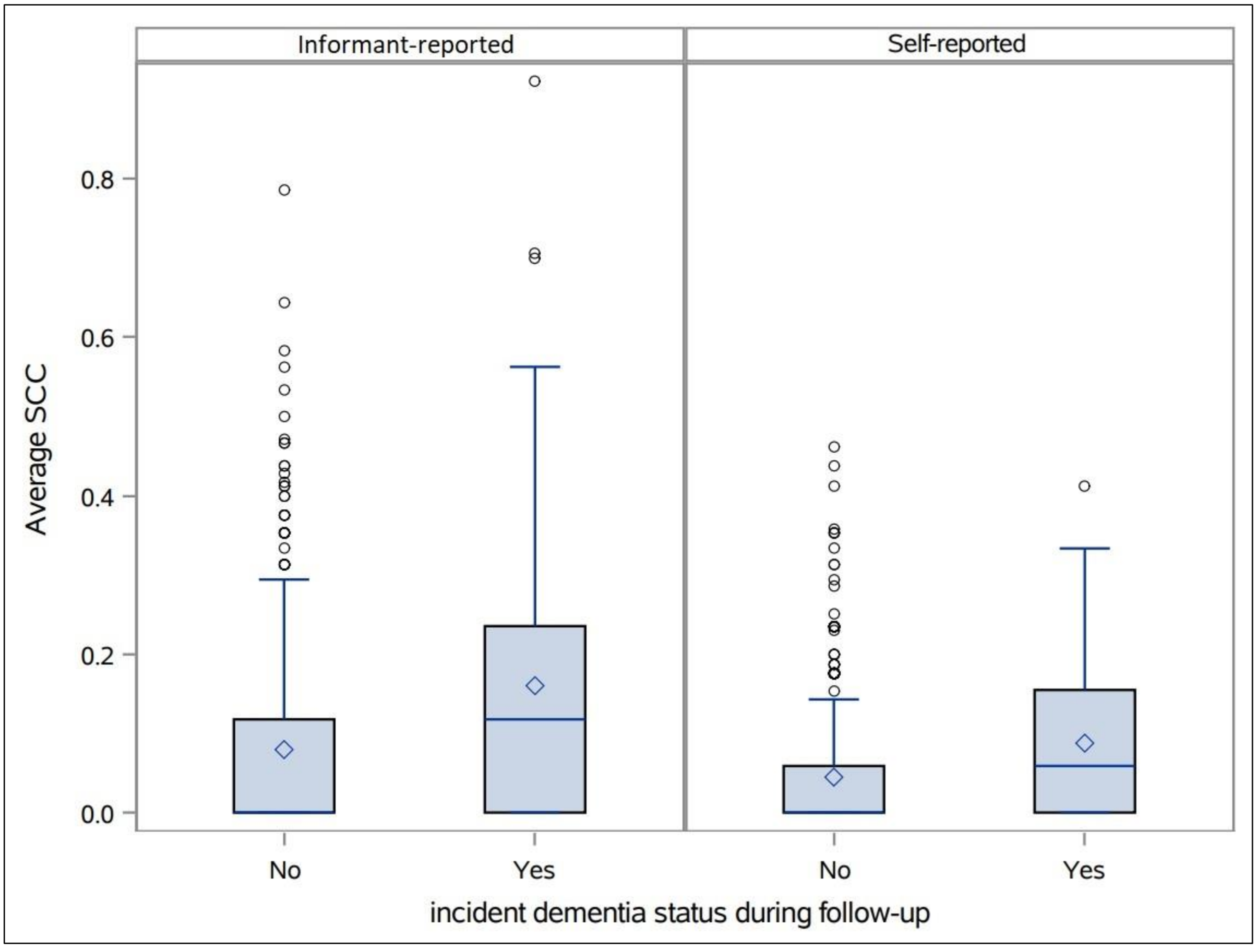

Supplementary Figure 3. Box plot of average self-reported and informant-reported SCC (measured with the CERAD questionnaire) by incident dementia status at follow-up in the subset of participants who had informants.
