# Supplementary material for: Examining the role of participant and study partner report in widely-used classification approaches of mild cognitive impairment in demographically-diverse community dwelling individuals: results from the Einstein aging study
Source: Front Aging Neurosci. 2023 Nov 21;15:1221768. doi: 10.3389/fnagi.2023.1221768 (PMC10702963; doi:10.3389/fnagi.2023.1221768)
Supplement: Supplementary file 2 [file Table_1.pdf]

**Supplementary Table 1.** Sampling of SCC measurement for MCI diagnosis

| | **Measure used** | **Source** | **Quantification of SCC** |
|---|---|---|---|
| Rapp et al., 2002 | Memory functioning questionnaire (MFQ) – assessing memory problems & mnemonic usage<br>64 items on 7 pt scale | Unspecified | Unspecified |
| van Uffelen et al., 2005 | Do you have memory complaints?<br>Cognitive scale of Strawbridge | Unspecified | Answering YES to "Do you have memory complaints?" or,<br><br>"Sometimes" at least twice on cognitive scale of Strawbridge |
| Baars et al., 2010 | Do you consider yourself forgetful? YES/NO<br>Worry & hindrance questions on 5-pt scale | Self-report | Score of 3 (out of 5) on either worry or hindrance questions or both |
| Potter et al., 2009 | IQCODE, 16-items about cognitive/functional change over the past 2 years<br>Rated on 5-pt scale | Informant-report | Not used to diagnose MCI |
| Smith et al., 2010 | 4 items on SCC from the Cambridge Examination for Mental Disorders; IQCODE | Self- and informant-report | Unspecified and not used to diagnose MCI |
| Ganguli et al., 2011 | Unspecified questionnaire | Unspecified | At least 2 items (median score) endorsed from 16 "remembering" questions and 5 "non-remembering" questions |
| Scherder et al., 2003; Thal et al., 2005; Chen et al., 2006; Winblad et al., 2004; Kinsella et al., 2009; Klekociuk & Summers, 2013; Sheikh et al., 2017; Dicks et al., 2018; Park et al., 2018; Knapstad et al., 2019; Kwok | Unspecified | Unspecified | Unspecified, but mention of Petersen or NIA-AA (Albert et al., 2011) criteria |

| | | | |
|---|---|---|---|
| et al., 2020; Broadhouse et al., 2021; Bastin et al., 2021 | | | |
| Gifford et al., 2015 | Clinical interview, no specific measures | Clinician-report | Unspecified, based on answers to interview questions |
| Balogh et al., 2022; Chandler et al., 2023 | None | N/A | SCC not used to diagnose MCi |
| Tsai et al., 2018; Li et al., 2018; Facal et al., 2019; Pusil et al., 2019; Leng et al. 2020; Gauthier, 2004; De Simone et al., 2023 | Unspecified | Self- & informant-report | Unspecified, self-SCC corroborated by informant |
| Jester et al., 2020; Sibilano et al., 2023 | Unspecified | Self- or informant-/clinician- report | Unspecified |
| Mazzeo et al., 2019 | Memory Assessment Clinics Questionnaire | Unspecified | Unspecified |
| Juncos-Rabadán et al., 2013 | Questionnaire for subjective cognitive complaints (QSCC), 7-items scored on a 5-pt Likert scale | Unspecified | "Evidence of concern about a change in cognition, in comparison with the previous level." |
| Hao et al., 2021 | SCD-Q9, 9 questions about overall memory function (5 questions) and time comparison (4 questions). Scored on a 3-pt Likert scale. | Self-report | Answering YES to "Do you have problem in memory?" |
| Nosheny et al., 2019 | ECog, 39 questions that measure changes in functional activities compared to 10 years ago using a 5-pt Likert scale. | Self- and informant-report | SCC not explicitly stated ass being measured for diagnosis of MCI |
